# Comparative Bio-Potential Effects of Fresh and Boiled Mountain Vegetable (Fern) Extract Mediated Silver Nanoparticles

**DOI:** 10.3390/plants11243575

**Published:** 2022-12-18

**Authors:** Gitishree Das, Han-Seung Shin, Jayanta Kumar Patra

**Affiliations:** 1 Research Institute of Integrative Life Sciences, Dongguk University-Seoul, Seoul 10326, Gyeonggi-do, Republic of Korea; 2 Department of Food Science and Biotechnology, Dongguk University-Seoul, Seoul 10326, Gyeonggi-do, Republic of Korea

**Keywords:** comparative, biological potential, fern, gosari, silver nanoparticles, antioxidant, α-glucosidase inhibition, cytotoxicity

## Abstract

This current investigation was designed to synthesize Ag nanoparticles (AgNPs) using both the fresh (Fbf) and boiled (Bbf) Korean mountain vegetable fern (named Gosari) extracts and make a comparative evaluation of its multi-therapeutic potentials. The screening of phytochemicals in the fern extract was undertaken. The synthesized fern-mediated silver nanoparticles are characterized and investigated for their bio-potential like α-glucosidase inhibition, antioxidant, and cytotoxicity prospects. The obtained AgNPs were characterized by the UV-Vis Spectra, SEM, EDS, XRD, FTIR, DLS, Zeta potential analysis, etc. The synthesis of the Fbf-AgNPs was very fast and started within 1 h of the reaction whereas the synthesis of the Bbf-AgNPs synthesis was slow and it started around 18 h of incubation. The UV-Vis spectra displayed the absorption maxima of 424 nm for Fbf-AgNPs and in the case of Bbf-AgNPs, it was shown at 436 nm. The current research results demonstrated that both Fbf-AgNPs and Bbf-AgNPs displayed a strong α-glucosidase inhibition effect with more than 96% effect at 1 µg/mL concentration, but the Bbf-AgNPs displayed a slightly higher effect with IC_50_ value slightly lower than the Fbf-AgNPs. Both Fbf-AgNPs and Bbf-AgNPs displayed good antioxidant effects concerning the in vitro antioxidant assays. In the case of the cytotoxicity potential assay also, among both the investigated Fbf-AgNPs and Bbf-AgNPs nanoparticles, the Bbf-AgNPs showed stronger effects with lower IC_50_ value as compared to the Fbf-AgNPs. In conclusion, both the fern-mediated AgNPs displayed promising multi-therapeutic potential and could be beneficial in the cosmetics and pharmaceutical sectors. Though the synthesis process is rapid in Fbf-AgNPs, but it is concluded from the results of all the tested bio-potential assays, Bbf-AgNPs is slightly better than Fbf-AgNPs.

## 1. Introduction

Nanotechnology has emerged as an important tool, which involves the synthesis or fabrication of large molecules (particles) in nanoscale sizes, called nanoparticles. Due to their numerous properties, such as their reactivity, physical properties (size in nanometer ranges), and their possible applications in disease diagnostics, and drug delivery, also in antimicrobial and antioxidant studies, the use of metal nanoparticles is beneficial [1]. It has become a central part of modern diagnostic and management technologies [2]. Owing to their unique physicochemical properties, silver nanoparticles (AgNPs) are among the most attractive nanomaterials in biomedicine. It is well-known for its biological activities, like promoting bone healing, repairing wounds, and enhancing the immunogenicity of vaccines broad-spectrum. It is highly useful in medical applications [3]. The bio-fabricated silver nanoparticles can be more reliable and helpful in disease diagnosis and prevention. In recent times, a number of applications of silver ions have been reported to be used in the management of food hygiene, water purification, and dental work for bacterial growth including catheter disinfection, etc. [4].

The synthesis of metal nanoparticles is mainly accomplished by the reduction of the metal salts in a mixture solution or accumulation of the atoms [5]. For nanoparticle synthesis, three different approaches are followed namely, physical, chemical, and biological approaches. However, there are associated harmful effects such as more energy consumption and toxic waste materials by using both the physical and chemical methods of synthesis. Therefore it is preferred to use the biogenic methods of nanoparticle synthesis (green synthesis) that are environmentally friendly, cost-effective, and above all, a one-step method [5,6,7]. In recent times, there is an increasing interest among scientists in using plants in the biosynthesis of nanoparticles due to their high bioactive potential, easy synthesis process, and nonpathogenicity [5,7]. It is reported that a crude plant extract contains plenty of phenolic compounds, flavonoids, alkaloids, etc., which can play a substantial part in the biosynthesis, covering, and equilibrium of the nanoparticles [8,9]. Considering this, the use of plant materials in the biosynthesis of silver nanoparticles could be an ideal option in the current investigation.

Ferns or mountain vegetables are the major divisions of the pteridophytes. Ferns have been reported to be rich in essential omega-6 and omega-3 fatty acids and antioxidant compounds [10,11]. The *P. aquilinum* contains amino acids, lignin, pectin, glucose, fructose, sucrose, ribose, caffeic acid, citric acid, aconite acid, minerals, vitamins like vitamin A, vitamin C, vitamin E, and B-Carotene, etc. [12]. The fern extracts were reported to have antimicrobial, antioxidant, antiviral, anti-inflammatory, and antiviral effects [13,14]. Concerning the pteridophytes-mediated green synthesis of AgNPs, only very few reports are available to date, so this much needs to be explored [15,16,17,18]. There is one report on the synthesis of Ag nanoparticles from the *P. aquilinum* leaf extract and its mosquitocidal and anti-plasmodial activity [19]. However, presumably, there is no report on the synthesis of silver nanoparticles (AgNPs) from both fresh and boiled *P. aquilinum* and no comparison of their biological activities to date. Therefore, in the current research, an effort has been made to synthesize the silver nanoparticles (AgNPs) using phytochemically rich fresh and boiled extracts of the mountain vegetable (fern) and investigate and compare their α-glucosidase inhibition, antioxidant, and cytotoxicity activities.

## 2. Results and Discussion

### 2.1. Biosynthesis and Characterization of Fbf and Bbf-AgNPs

Silver metals have high surface plasmon resonance (SPR), which is important in nanoparticle synthesis. There is an increasing demand for green synthesis of nanoparticles using biocompatible sources as it is safe, non-toxic, and economical [20]. Additionally, there is information on the toxic effect of biosynthesized AgNPs [21], so it is necessary to ensure a safety survey in depth that, bio-synthesized nanoparticles for a human being are out of side effects.

Application of plants and their extracts for the green biosynthesis of nanoparticles offers a wide-ranging benefit over different synthesis techniques as it is cost-effective, environmentally friendly, and can offer large-scale manufacture of nanoparticles. Silver nanoparticles’ green synthesis, using plant extracts holding phytochemicals or bioactive compounds has received considerable attention, as the Ag nanoparticles have various medicinal and other applications [22]. Biosynthesis of Fbf-AgNPs and Bbf-AgNPs was attained in this research, by using fresh and boiled mountain vegetables or *P. aquilinum* fern aqueous extracts. In the beginning, to identify the presence of phytochemicals or bioactive compounds, the phytochemical analysis of the fresh and boiled extracts of Fbf and Bbf was carried out. In both Fbf and Bbf extracts, phytochemicals like flavonoids, saponins, and carbohydrates are found to be commonly present. Whereas some other phytochemicals were also found which were not common in both extracts (Table 1).

**Figure 1:**
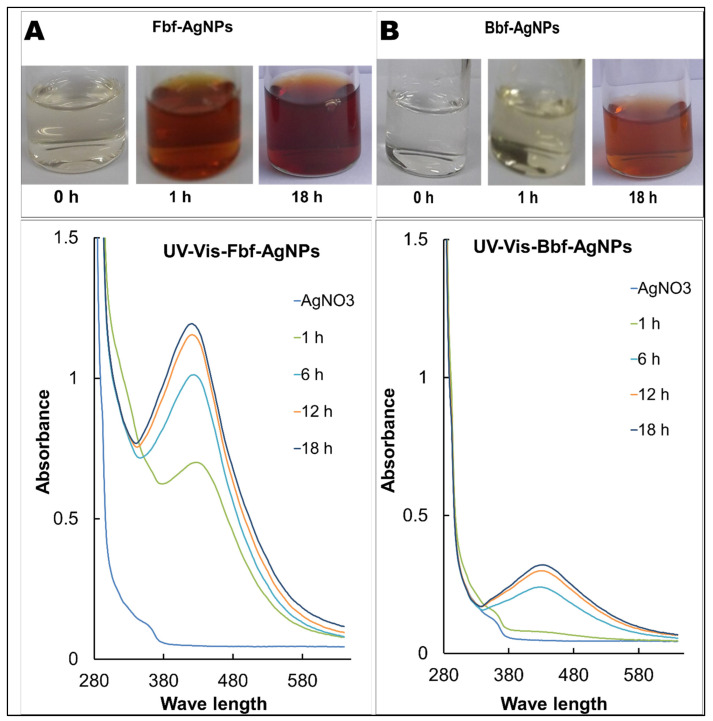
The UV-Vis spectra of the biosynthesized nanoparticles showing the SPR. (**A**) UV-Vis spectra of Fbf-AgNPs and (**B**) UV-Vis spectra of Bbf-AgNPs. Inset, visible color changes in the reaction mixture at different time.

**Figure 2:**
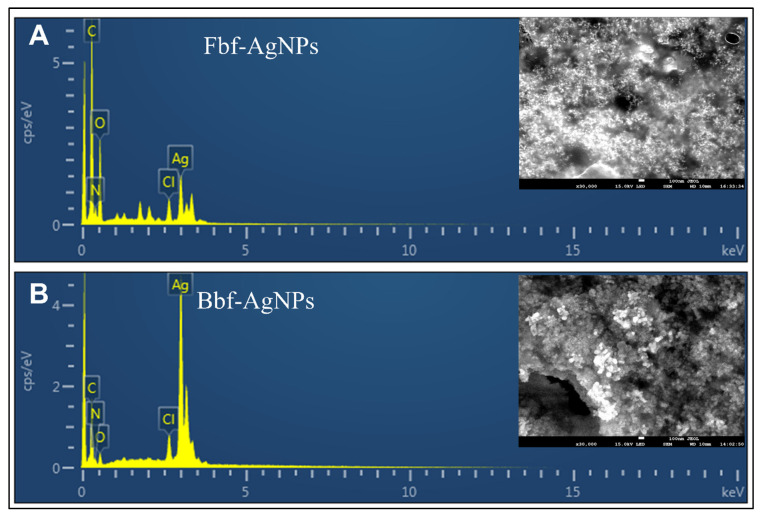
The SEM and EDX spectra of the biosynthesized nanoparticles showing the morphology and elemental composition. (**A**) Fbf-AgNPs and (**B**) Bbf-AgNPs.

In terms of the change of pigment of the test solution from colorless to brown color, biosynthesis of both Fbf and Bbf-AgNPs was proven by visual assessment (Figure 1A) [23]. After visual confirmation of the test solution of Fbf, and Bbf-AgNPs, it was analyzed under a UV-Vis spectrophotometer. The absorbance value was constantly monitored at regular intervals up to 18 h. The SPR value of the Fbf-AgNPs was found to as 424 nm whereas the SPR value of the Bbf-AgNPs was found to as 436 nm (Figure 1B), which also confirms the biosynthesis of both Fbf-AgNPs and Bbf-AgNPs. This result corroborates with the previously published nanoparticle synthesis results [24]. A change in the SPR of Bbf-AgNPs prepared with the Bbf extract was observed compared to that of the Fbf-AgNPs prepared with the Fbf extract might be due to the blue shift due to the decrease in the refractive index of the dielectric environment surrounding the synthesized nanoparticles [25,26]. The basic morphology, characteristics, and elemental composition of both the Fbf-AgNPs and Bbf-AgNPs were estimated by SEM and EDX assessment. The SEM study results of both biosynthesized Fbf-AgNPs, and Bbf-AgNPs were based on nanometer-scale imaging (Figure 2A). Similar results were also mentioned in earlier reports [27,28]. The biosynthesized Fbf-AgNPs and Bbf-AgNPs elemental arrangement was known by the EDS analysis, and it showed the presence of nanoparticles (Ag). The EDS results of the Fbf-AgNPs and Bbf-AgNPs identified the presence of the element (Ag) (Figure 2B). The present results are analogous to previous research reports [29].

**Figure 3:**
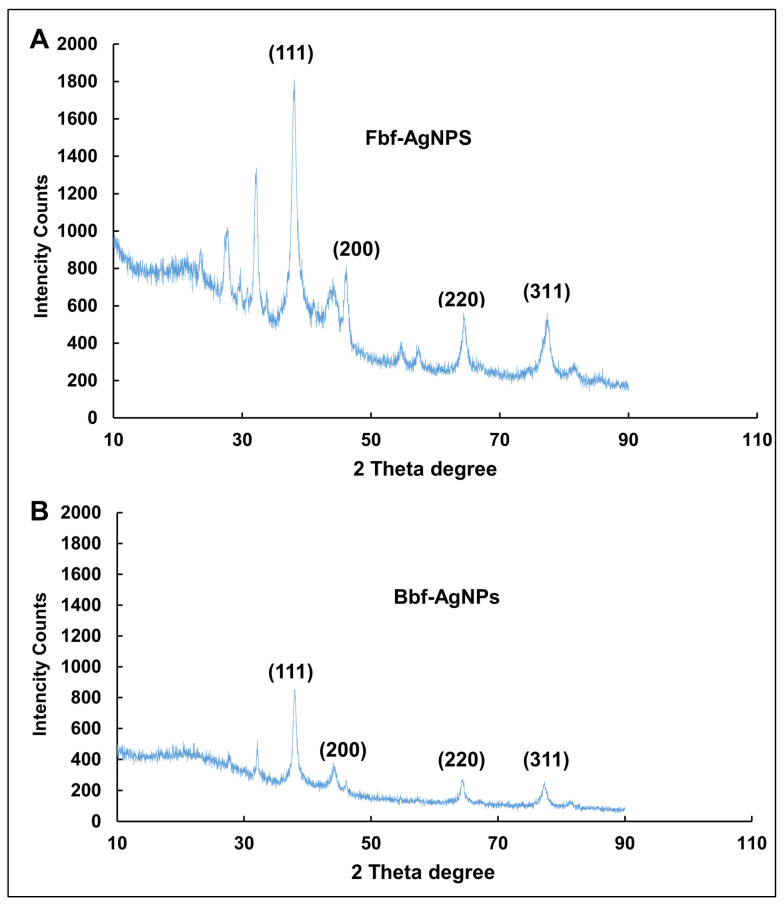
XRD spectra of the biosynthesized nanoparticles. (**A**) Fbf-AgNPs and (**B**) Bbf-AgNPs.

The XRD showed the physical structure and nature of the generated nanoparticles. From the XRD peaks, it was clear that the Fbf-AgNPs and Bbf-AgNPs were crystalline. The peak values were equivalent to the Ag0 standard JCPDS card no. 04-0783(fccp.) [29] (Figure 3). In the case of Fbf-AgNPs, the diffraction peaks were visible at 38.06, 46.18, 64.51, and 76.95 which are equivalent to (111), (200), (220), and (311) as per the JCPDS card no. 04-0783 (Figure 3A). However, in the case of Bbf-AgNPs, the diffraction peaks were detected at 38.01, 46.02, 64.64, and 76.88 which were also equivalent to (111), (200), (220), and (311), detailed and presented in Figure 3. Apart from the specified peaks, few other peaks were observed in both cases, which might be due to the phytochemicals or bioactive compounds prevailing in the Fbf and Bbf extracts that might have acted as both reducing and capping agents during biosynthesis of Fbf-AgNPs and Bbf-AgNPs [30,31,32,33]. Similar results on the XRD peaks of the biosynthesized silver nanoparticles are also presented in the previously published literature [20,29,34].

**Figure 4:**
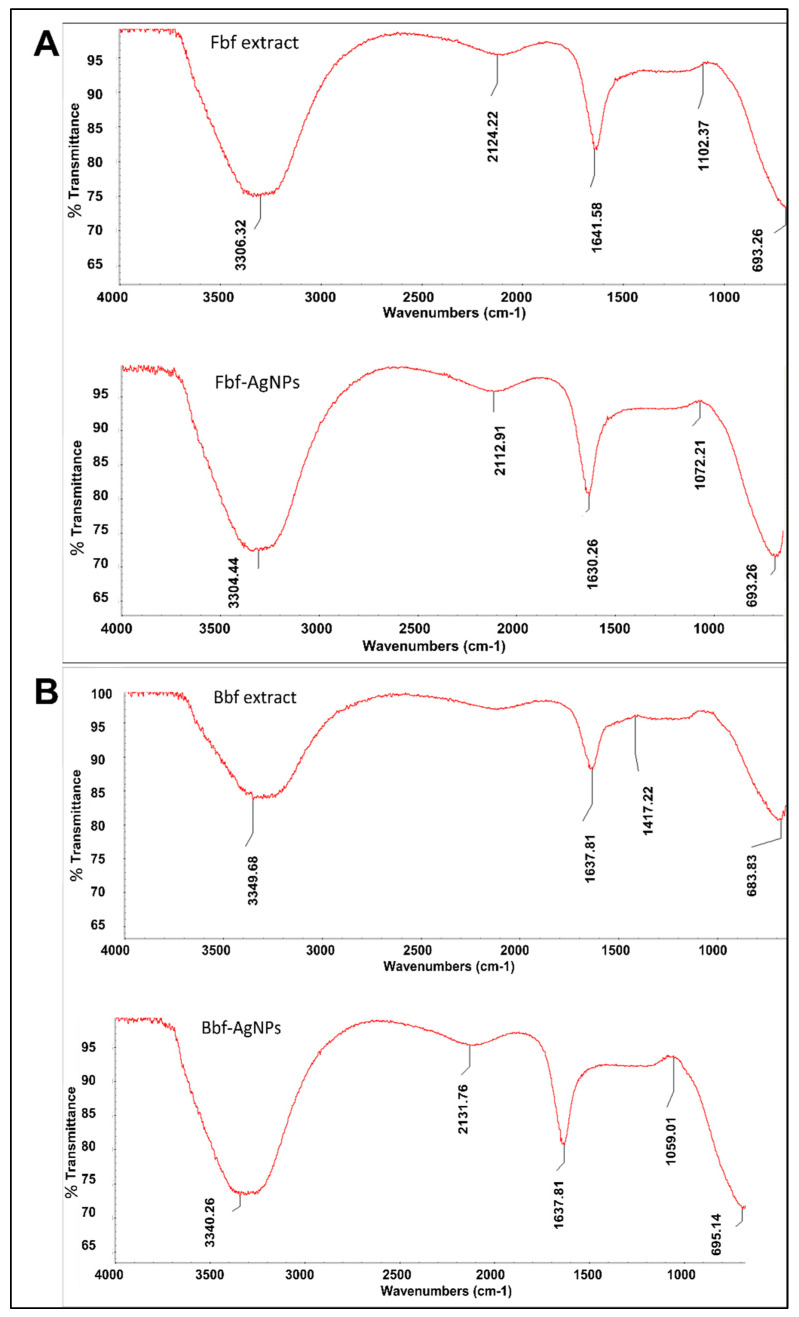
FTIR analysis of the biosynthesized nanoparticles and the fern extracts. (**A**) Fbf extract and Fbf-AgNPs and (**B**) Bbf extract and Bbf-AgNPs.

By FTIR analysis, the main functional groups present in the Fbf-AgNPs, Bbf-AgNPs, and the Fbf and Bbf extracts were identified. The shift of peak values observed in the synthesized AgNPs suggests their active contribution to the fabrication of Ag nanoparticles. FTIR results displayed different stretching modes with different peak standards (Figure 4A). The Fbf extract peak standards were observable at 3307.38, 2124.22, 1641.58, 1102.37, and 693.26 cm^−1^. These numbers were possibly shifted to 3304.44, 2112.91, 1632.19, 1072.21, and 693.26 cm^−1^, respectively, in the case of Fbf-AgNPs, whereas in the case of Bbf extract, the peak value that was visible at 3349.68, 1637.81, 1417.22, and 683.83 cm^−1^ probably shifted to 3340.26, 1637.81, 1059.01, and 695.14 cm^−1^, correspondingly, in Bbf-AgNPs (Figure 4B).

For Fbf-AgNPs, the peak at 3304.44 cm^−1^ states the O-H bond, and H-bonding stretching, which comes under functional groups, alcohols, and phenols [35]. The next peak at 2112.91 cm^−1^ postulates the presence of the C≡N bond, of nitrile groups. The 3rd peak at 1632.19 cm^−1^ postulates the N-H bonds of the primary amine functional group. The 4th resultant peak value at 1072.21 cm^−1^ stipulates the C-N stretch bond of the aliphatic amines functional groups. The last resultant peak at 693.26 cm^−1^ signifies the -C≡C-H: C-H bend bond of alkynes functional groups.

For Bbf-AgNPs, the peak values at 3340.26 cm^−1^ designate the N-H stretch bond of 1°, 2° amines, and amides functional group. The second peak at 1637.81 cm^−1^ specifies, the presence of the C=O stretch bond of α, β-unsaturated aldehydes, and ketones. The 3rd peak at 1059.01 cm^−1^ specifies the presence of the C-O stretch of alcohols, carboxylic acids, esters, and ethers groups. The last peak at 695.14 cm^−1^ states the presence of a strong =C-H bend bond of the alkenes group [35]. The change in the observed peak value of the Fbf extract and Fbf-AgNPs, Bbf extract, and Bbf-AgNPs could be responsible for the stability and outer coating of both the generated nanoparticles [29]. The analogous consequence has been stated in the previous investigation [36].

**Figure 5:**
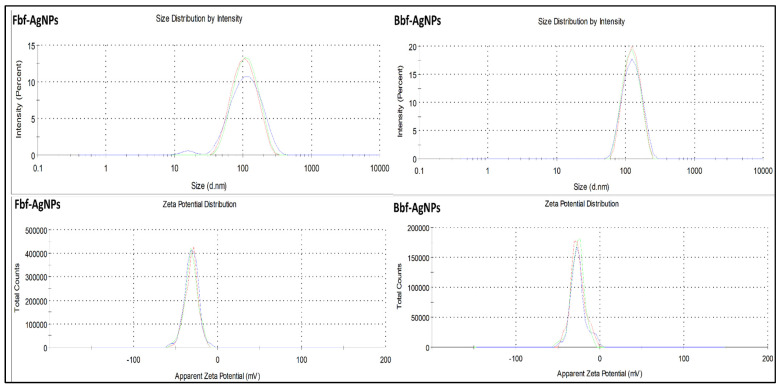
Size distribution and Zeta potential of the biosynthesized Fbf-AgNPs and Bbf-AgNPs.

The Fbf-AgNPs and Bbf-AgNPs hydrodynamic diameter or distribution of size and surface charges were evaluated through DLS and Zeta potential study. The hydrodynamic diameter or size distribution (average size) of Fbf-AgNPs and Bbf-AgNPs is 91.09 d.nm and 122.9 d.nm, respectively, with the PDI value of 0.233 mV and 0.301 mV (Figure 5). This confirms that both the synthesized nanoparticles are in the nanometer range. Similar results were stated in previously published research articles [37]. The zeta potential of Fbf-AgNPs, and Bbf-AgNPs was highly negative, i.e., −31.1 and −26.3, respectively (Figure 5). The highly negative charge supports the long time stability of the synthesized AgNPs [38,39]. It also confirmed the colloidal nature of both the synthesized nanoparticles [40]. This result is similar to earlier published articles [41,42,43].

### 2.2. Comparative Study of the Bio-Potential Effect of Fbf-AgNPs and Bbf-AgNPs

#### 2.2.1. α-Glucosidase Inhibition Potential of Fbf-AgNPs and Bbf-AgNPs

**Figure 6:**
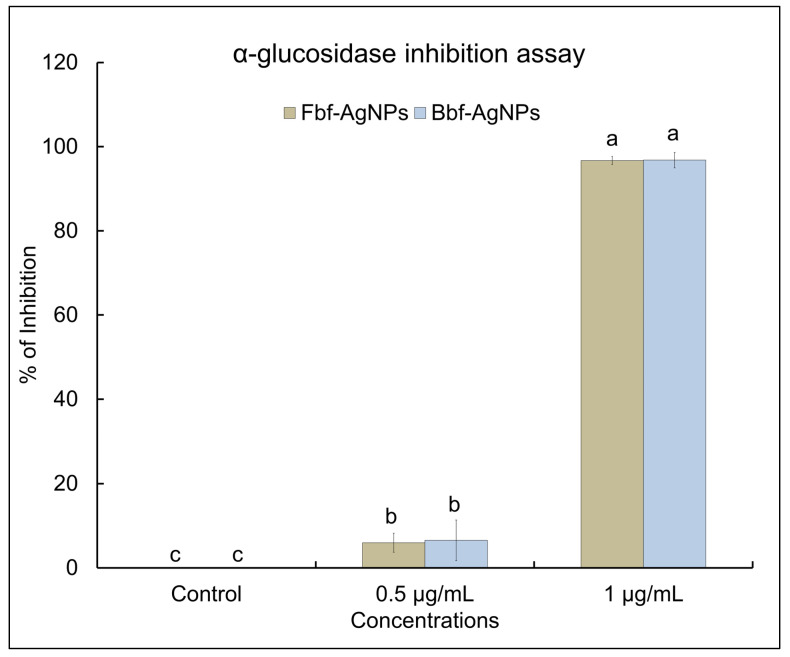
α- glucosidase inhibition potential of the biosynthesized Fbf-AgNPs and Bbf-AgNPs. Differences in superscript letters indicate statistically significant differences at *p* < 0.05.

The obtained Fbf-AgNPs and Bbf-AgNPs were investigated for their α-glucosidase inhibition potential. Both AgNPs showed a significant α-glucosidase inhibition effect. At two different tested concentrations of 0.5 µg/mL and 1.0 µg/mL, the Bbf-AgNPs showed a slightly higher α-glucosidase inhibition effect than the Fbf-AgNPs (Figure 6). In the case of 0.5 µg/mL concentration, the Fbf-AgNPs displayed (5.97%) inhibition, whereas the Bbf-AgNPs displayed slightly higher (6.5%) inhibition at the same concentration. At the highest tested concentration (1.0 µg/mL), both the generated AgNPs displayed maximum inhibition with values of more than 90%. The Fbf-AgNPs displayed 96.71% inhibition (at 1.0 µg/mL concentration) whereas the Bbf-AgNPs displayed 96.80% inhibition (at 1.0 µg/mL concentration). The calculated IC_50_ value of Bbf-AgNPs and Fbf-AgNPs was determined as 1.44 µg/mL and 1.56 µg/mL, respectively, which indicates that the Bbf-AgNPs have a slightly higher α-glucosidase inhibition effect than that of the Fbf-AgNPs (Table 2). Both synthesized AgNPs displayed better α-glucosidase inhibition potential at lower concentrations than earlier reported biosynthesized nanoparticles [18,44]. It is believed that the phytochemicals present in the Fbf and Bbf extracts that performed as the reducing agent in the biosynthesis of the nanoparticles, could have also acted as the capping agents and might have been present in the surface of the nanoparticles that are responsible for its strong α-glucosidase inhibition potential. A similar assumption is also provided in the previously published literature [45,46].

#### 2.2.2. Cytotoxicity Study of Fbf-AgNPs and Bbf-AgNPs

**Figure 7:**
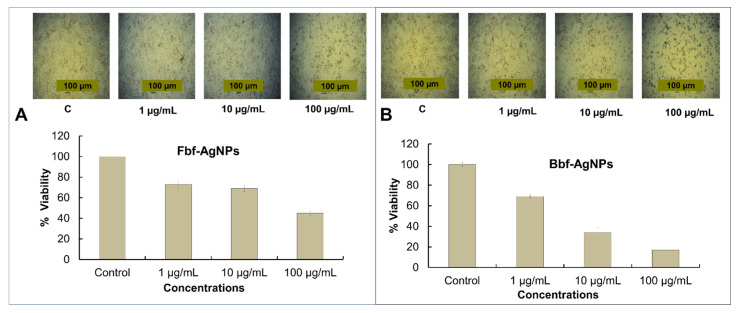
Cell viability percentage and the morphology of the biosynthesized Fbf-AgNPs and Bbf-AgNPs at different concentrations. Inset: Images (**A**,**B**) of the cell viability at different treated concentrations.

Nano-biotechnology is an energetic scope of science that exhibits possible application in cancer therapy, molecular imaging, molecular diagnosis, targeted chemotherapy, etc. [47]. The significance of ferns has been established previously and the medicinal usage such as anticancer, antidiabetic, and antioxidant potentials, etc. of several Pteridophytes was already reported [48]. As cancer claims millions of individual lives yearly, the development of therapeutics from natural or biological sources for treating cancer is increasing significantly [49]. In controlling cancer-related conditions, nano-medications have been acknowledged to be extremely effective [50,51]. In this investigation, the biosynthesized Fbf, and Bbf-AgNPs exhibited significant cytotoxicity potential. Both Fbf-AgNPs and Bbf-AgNPs displayed dose-dependent cytotoxicity activity. The cytotoxicity effect of the silver nanoparticles might be affected by many things such as shape, size, and configuration of nanoparticles. The change in the above things may change the cytotoxicity effect [52,53]. For biomedical uses biosynthesized NPs are desired which are eco-friendly and toxic-free [54]. In this research, in the cell viability study as the concentration of the tested AgNPs decreased, it was detected that the HepG2 live cells increased, and the cell death decreased (Figure 7). The morphology of HepG2 cancer cell lines treated with different concentrations of Fbf-AgNPs and Bbf-AgNPs was observed under an inverted light microscope. Both synthesized AgNPs showed effective cytotoxicity potential. The Fbf-AgNPs and Bbf-AgNPs at 100 µg/mL were significantly toxic to the treated HepG2 cancer cell lines. The alive cell lines percentage escalates when the concentration of both the treated Fbf-AgNPs and Bbf-AgNPs was reduced (Figure 7). The cytotoxicity potential showed slightly higher in the case of Bbf-AgNPs in comparison with Fbf-AgNPs at maximum (100 µg/mL) tested concentration as the viability or live HepG2 cancer cells percentage was lower (17%) in the case of Bbf-AgNPs, whereas it was slightly higher (45%) in case of Fbf-AgNPs (Figure 7). Additionally, the IC_50_ value of Bbf-AgNPs was lower which showed that the Bbf-AgNPs have slightly higher effectiveness than Fbf-AgNPs (Table 2). The cytotoxicity potential of both the obtained AgNPs was higher or parallel with earlier reported results [53,55]. It was also reported earlier that the electrostatic pull amid nanoparticles and the cells might be important for the existing cytotoxicity of the Ag nanoparticles against the cancerous cells [56]. The phytochemicals present in the Fbf and Bbf extracts, which are capped on the nanoparticle surface, could have been responsible for their strong cytotoxicity potentials. These results corroborate previously published results, which reported that nanoparticles have broad-spectrum anticancer effect [57].

#### 2.2.3. Antioxidant Study of Fbf-AgNPs and Bbf-AgNPs

**Figure 8:**
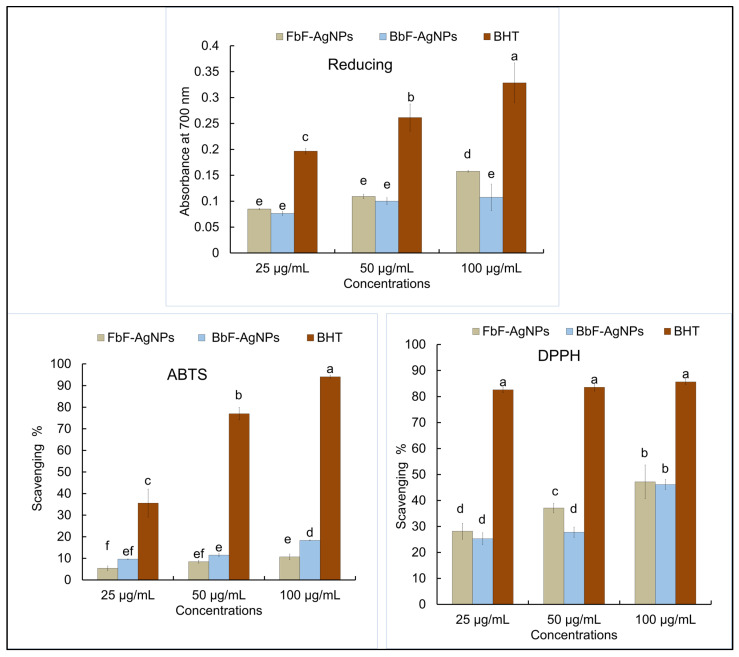
In vitro antioxidant potential (reducing power, ABTS and DPPH assay) of Fbf-AgNPs and Bbf-AgNPs. Differences in superscript letters in each assay indicate statistically significant differences at *p* < 0.05.

**Figure 9:**
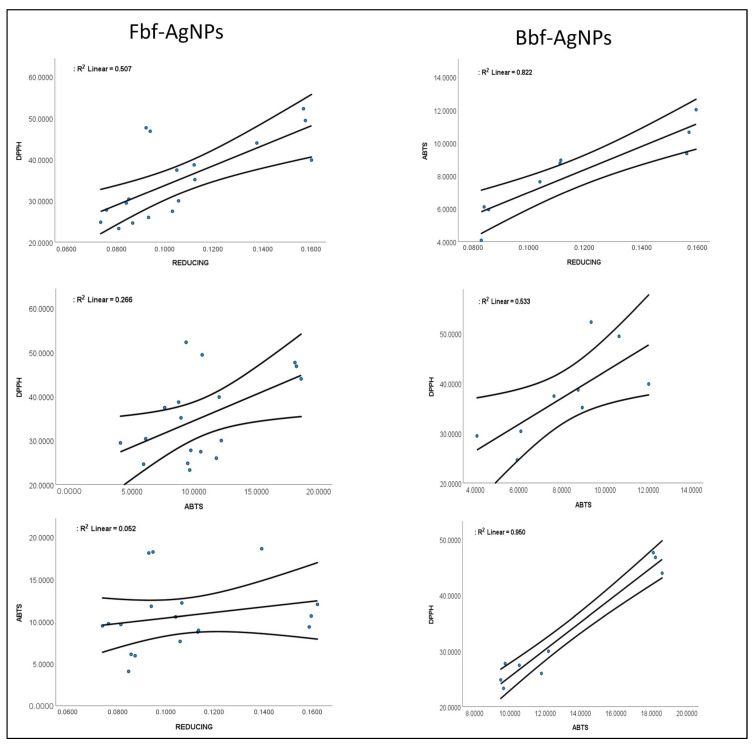
Correlation curves between the different antioxidant parameters (reducing power, ABTS and DPPH assay) of Fbf-AgNPs and Bbf-AgNPs.

Further, both the Fbf-AgNPs, and Bbf-AgNPs were investigated for their antioxidant effects such as reducing power, scavenging of the DPPH (1,1-diphenyl-2-picrylhydrazyl), and ABTS ([2,2′-azino-bis(3-ethylbenzothiazoline-6-sulphonic acid)]) free radicals at altered concentrations (25, 50, and 100 µg/mL), and the effects are presented in Figure 8. The study results displayed concentration dependency that increases with the upsurge in the concentration of the treated Fbf-AgNPs, and Bbf-AgNPs. In the reducing study, the Fbf-AgNPs showed a slightly higher effect than Bbf-AgNPs at 100 µg/mL concentration. In the case of the DPPH assay, the Fbf-AgNPs also showed a slightly higher scavenging percentage than the Bbf-AgNPs at 100 µg/mL concentration. However, in the ABTS assay, the Bbf-AgNPs showed a slightly higher scavenging percentage than the Fbf-AgNPs at 100 µg/mL concentration) Figure 8. Overall, both the Fbf-AgNPs and Bbf-AgNPs showed moderate antioxidant scavenging potential. However, Fbf-AgNPs showed a slightly higher effect than that of the Bbf-AgNPs in reducing power and DPPH assays. Both the Fbf-AgNPs and Bbf-AgNPs displayed moderate antioxidant potentials which are comparable to the earlier reported results [44,58]. The IC_50_ or the IC_0.5_ values of the three antioxidant assays are presented in (Table 2). For reducing power assay, the IC_0.5_ value is 231.07 µg/mL for Fbf-AgNPs, and 292.66 µg/mL for Bbf-AgNPs, and BHT (Butylated hydroxytoluene) the IC_0.5_ value was 103.82 µg/mL. In the case of the DPPH assay, the IC_50_ values of Fbf-AgNPs and Bbf-AgNPs were 72.59 µg/mL and 82.56 µg/mL, respectively, and for BHT, it was 34.46 µg/mL. In the case of the ABTS assay, the IC_50_ values of Fbf-AgNPs and Bbf-AgNPs were 332.40 µg/mL and 206.68 µg/mL, respectively, and for BHT, it was 40.24 µg/mL (Table 2). Further, the correlation curves between the different antioxidant parameters of both the Fbf-AgNPs and Bbf-AgNPs are plotted against each other (Figure 9). The results showed a more significant positive trend between the antioxidant parameters of Bbf-AgNPs as compared to that of Fbf-AgNPs with the r^2^ value of 0.950 between the DPPH and ABTS assay. It is conferred that the Bbf-AgNPs might possess more antioxidant potential and protection against the reactive oxygen species [59]. The bioactive compounds or phytochemicals like flavonoids, phenolics, and other active components present in the Fbf and Bbf extracts that played a momentous role in the outer covering and steadiness of the synthesized nanoparticles could also have significantly participated in the existing antioxidant potential of both nanoparticles [57,60]. Besides, the antioxidant potential of both the Fbf-AgNPs and Bbf-AgNPs could mainly be accredited to the redox potential of the phenolic compounds present in the Fbf and Bbf extracts that were used as the reducing agent in the biosynthesis of the nanoparticles [61]. The acquired results could prove the hypothesis that the capping of Fbf and Bbf extracts on the surface of the nanoparticles might have enhanced their antioxidant properties.

## 3. Materials and Methods

### 3.1. Plant Materials and Preparation of Extracts

The fresh and boiled mountain vegetables or *P. aquilinum* fern, i.e., termed Fbf and Bbf, respectively, were obtained from the local market. The commercial boiled fern was prepared by completely drying the fresh fern followed by soaking them in room temperature water for 12 h, then boiling for 5 min, and then the fern is packaged and sold to markets.

For preparing the plant extracts, both these samples after collecting were, rinsed properly, dried using tissue paper, and cut into tiny pieces. About 50 g of each sample were transferred to two separate glass flasks (1 Lt) with 250 mL double distilled water, boiled for approximately 30 min with continuous stirring, cooled down and sieved, and the liquid sample was stored in an airtight container for use.

### 3.2. Primary Phytochemical Study of Fbf and Bbf Extracts

The primary phytochemical study of the Fbf and Bbf extracts was done to investigate the occurrence of a variety of bioactive compounds or phytochemicals such as carbohydrates, tannin, terpenoids, flavonoids, saponins, steroids, and cardiac steroidal glycoside, etc. Earlier standard conventional procedures were followed with slight modifications to carry out the experiments. For the flavonoids test: 1 mL of both Fbf and Bbf extracts in two separate test tubes were taken and to them, and 1 mL of diluted NaOH was added. The appearance of gloomy precipitation confirms the presence of the flavonoid [62]. In the case of the carbohydrate test: two test tubes were taken with 2 mL of extract from each. Next, Molisch’s reagent (5% 1-naphthol in alcohol, 2 drops) was added and mixed by shaking. Around 1 mL of the concentrated H_2_SO_4_ was added slowly in the test tubes towards its side wall. The formation of a red-violet or purple ring at the middle junction of both the liquid layers stipulates the presence of carbohydrates [63].

For the Tannin test: 5 mL of the filtered extracts of both Fbf and Bbf were taken in two separate test tubes and a few drops of FeCl_3_ (0.1%) were added to them. The appearance of brownish-green or bluish-black color indicates the existence of tannin. In the case of the terpenoids test: the extracts of Fbf and Bbf (5 mL) were taken in two test tubes and 2 mL of chloroform was mixed with them followed by the addition of concentrated sulfuric acid (3 mL). The appearance of a reddish brown color interface confirmed the presence of terpenoids [64]. For the saponins test, the 2 g sample of both Fbf and Bbf were boiled in 20 mL of double distilled water using a hot water bath and were then filtered. Next, 5 mL of the filtrated sample was mixed with 2.5 mL of double distilled water and vigorously shaken for a steady persistent froth. On the froth, a few drops of olive oil was added, again shaken vigorously, and then the development of emulsion which confirmed the existence of saponins was witnessed [65]. The steroid test was carried out by following the standard protocol with slight alteration [63]. Both Fbf and Bbf extract (1 mL each) were added in two test tubes, and to them, around 2 mL each of acetic anhydride and sulfuric acid were carefully poured one after the other and observed for color change. The appearance of violet-blue or green color indicates the existence of steroids. The cardiac steroidal glycoside test was done by following the protocol of [66] with slight variation. A solution of 4 mL of glacial acetic acid with a drop of 2%Fecl_3_ was mixed with 10 mL each of Fbf and Bbf sample extract and concentrated H_2_SO_4_ (1 mL). A brown ring formed in the middle of the layers confirmed the presence of cardiac steroidal glycosides.

### 3.3. Biosynthesis of Fbf and Bbf Extracts-Mediated AgNPs

The biosynthesis of Fbf-AgNPs and Bbf-AgNPs was attained by using the fresh and boiled mountain vegetable *P. aquilinum* fern as the only reducing agent in the reaction, by following the standard protocols [67,68] with slight variation. In brief, in two separate 250 mL conical flasks, AgNO_3_ (1 mM, 80 mL) and Fbf and Bbf extracts (20 mL each) were mixed separately and slowly with continuous stirring. The synthesis of Fbf-AgNPs and Bbf-AgNPs was examined by observing the variation in the coloration of the mixture. The reaction was allowed to continue till 24 h. After that, the synthesized Fbf-AgNPs and Bbf-AgNPs were collected and centrifuged for 30 min (at 12,000 rpm), followed by 3–4 times washing with distilled water in order to remove any unbound Fbf and Bbf extracts. The collected palate was dried at 40 °C and stored in airtight containers until further characterization.

### 3.4. Characterizations of Bio-Synthesized Fbf-AgNPs and Bbf-AgNPs

The characterization of Fbf-AgNPs and Bbf-AgNPs after synthesis was done by following different standard analytical methods like UV-Vis spectroscopy, SEM, EDS analysis, XRD analysis, size, FT-IR, and zeta potential study, etc. by following the previously stated standard protocols [67,69,70,71]. The Ag^+^ ion reduction to form Fbf-AgNPs and Bbf-AgNPs was studied by assessing the reaction solution absorption spectra through a UV-VIS spectrophotometer (Thermo Scientific, Waltham, MA, USA). The Fbf-AgNPs and Bbf-AgNPs preliminary synthesis were established as a result of UV-VIS spectrophotometric scanning for 24 h in between 300–700 nm. The nature of synthesized Fbf-AgNPs and Bbf-AgNPs was described through the XRD analyzer at a setup of CuKα radians at 30 kV and 40 mA at 2θ angle (Panalytical, Almelo, The Netherlands). The powdered samples (Fbf-AgNPs and Bbf-AgNPs) were uniformly placed on the glass slide sample holder. In the XRD machine, the sample holder was positioned properly and scrutinized using the inbuilt software. The synthesized Fbf-AgNPs and Bbf-AgNPs morphology was investigated by the SEM analyzer (Hitachi S-3000N, Tokyo, Japan). On the sample holder, both of the samples were evenly spread and sputter coated in a platinum ion coater for 120 sec. Next, the elemental configuration of Fbf-AgNPs and Bbf-AgNPs was determined by using an EDS analyzer (EDAX Inc., Mahwah, NJ, USA) linked with the SEM. The Fbf extract, Fbf-AgNPs, Bbf extract, and Bbf-AgNPs FTIR spectra were evaluated through the FTIR spectrophotometer (ThermoFisher Scientific, Waltham, MA, USA), at 400 to 4000 cm^−1^ wavelengths for investigating the existence of the related functional groups contributing the synthesis of nanoparticles. The samples/2 µL of Fbf extract, Fbf-AgNPs, Bbf extract, and Bbf-AgNPs were placed one by one separately at the instrument receiver and analyzed through various modes of vibration in computer software attached to the FTIR instrument. The Fbf-AgNPs and Bbf-AgNPs dynamic light scattering (size distribution) and zeta potential analysis were carried out by using a Zetasizer machine (Malvern Zetasizer, Malvern, WR14 1XZ, UK) as per the standard established protocol of Clogston and Patri [72]. The data were analyzed using the zetasizer software.

### 3.5. α-Glucosidase Inhibition Potential of Fbf-AgNPs and Bbf-AgNPs

The α-glucosidase inhibition effect of Fbf-AgNPs and Bbf-AgNPs was assessed by using the standard methods with slight variation [73,74]. Briefly, 50 µL of the mixture solution contains Fbf-AgNPs and Bbf-AgNPs (10 µg/mL), sodium phosphate buffer (0.02 M, pH 6.9) was aliquoted to a 96 well plate successively. To this mixture solution, 50 µL of α-glucosidase (0.5 U/mL concentration) was mixed. Then, it was kept at room temperature for 10 min. Next, 50 µL of P-nitrophenyl-glucopyranoside (3 mM concentration), used as substrate was added to it. The solution was rested for another 20 min at 37 °C followed by the addition of sodium carbonate (50 µL of 0.1 M), and the reaction was stopped. By using a microplate reader, the optical density (the value) of the reaction solution (test solution) was measured at 405 nm wavelength. The α-glucosidase inhibition was calculated as mentioned in the equation.

(1) $$Percentage\;effect=\frac{\left(C_{g}-T_{g}\right)}{C_{g}}\times 100$$

where *C_g_* is the value (control absorbance value) and *T_g_* is the value of (tested sample absorbance value).

### 3.6. Antioxidant Effect of Fbf-AgNPs and Bbf-AgNPs

The antioxidant effects of Fbf-AgNPs and Bbf-AgNPs were estimated through the reducing power assay, DPPH scavenging assay, and ABTS free radical scavenging assays using standardized methods [75]). In the reducing power assay, the reaction mixture consists of 50 µL of Fbf-AgNPs, Bbf-AgNPs, and the BHT at three concentrations ranging between 25–100 µL separately followed by the addition of 50 µL each of phosphate buffer (0.2 M, pH 6.6), and potassium ferricyanide (1%). The whole solution was mixed properly and incubated in dark at 50 °C for 20 min. Next, 10% trichloroacetic acid (50 µL) was mixed with the solution to dismiss the progression of the reaction. Next, it was centrifuged for 10 min at 3000 rpm and the supernatant (50 µL) was moved to a 96-well microplate, and to it, DD water (50 µL) and 0.1% FeCl_3_ solution (10 µL) were mixed, followed by further 10 min incubation at room temperature. The absorbance value was recorded at 700 nm wavelength to determine the reducing power.

The DPPH scavenging properties of Fbf-AgNPs, Bbf-AgNPs, and BHT were estimated using standard procedures. The reagent solution of DPPH (0.1 mM) and Fbf-AgNPs, Bbf-AgNPs, and BHT (25, 50, 100 µL) was prepared before starting the test. The reaction was started by mixing 50 µL each of the DPPH solution and Fbf-AgNPs, Bbf-AgNPs, and BHT in a microplate. The solution was mixed properly for 30 min under a dark condition with continuous shaking. After the incubation, the absorbance value of the reaction solution was estimated at 517 nm using a UV-VIS spectrophotometer. The DPPH scavenging potential of Fbf-AgNPs, Bbf-AgNPs, and BHT was calculated from Equation (1) and expressed as a percentage of DPPH scavenging.

For the ABTS radical scavenging action, a typical established method was followed. In brief, initially, the ABTS stock solutions were prepared by taking ABTS (7.4 mM) and potassium persulfate (2.6 mM) separately and incubated for 12 h and equally mixed just before the experiment. A total of 150 µL of the mixture solution contains, Fbf-AgNPs, Bbf-AgNPs, and BHT (15 µL, at (25, 50, and 100 µg/mL), respectively), and 135 µL of the ABTS working solution. Next, the reaction solution was kept in the dark for 2 h until the end of the scavenging response. This was followed by measuring the absorbance value at 750 nm and calculating the ABTS scavenging potential as per Equation (1).

Further, the effective concentration that exhibited 50% activity for all three assays was calculated as the IC_50_/IC_0.5_ values and recorded.

### 3.7. Preliminary Cytotoxicity Effect Assessment of Fbf-AgNPs and Bbf-AgNPs

The preliminary cytotoxicity effect of both the Fbf-AgNPs and Bbf-AgNPs against the HepG2 cancerous cell lines was evaluated as per the standard procedure reported earlier [76]. Before the experiment, both the Fbf-AgNPs and Bbf-AgNPs were dissolved individually in Dulbecco’s phosphate-buffered saline (Welgene, Gyeongsanbuk-do, Republic of Korea) (1 mg/mL) and sterilized with 0.22-micron syringe micro filter. Further, different dilutions of the NPs were prepared in complete Dulbecco’s Modified Eagle Medium (DMEM) supplemented with penicillin-streptomycin (1%) and Fetal Bovine Serum (10%) for the treatment of HepG2 cells. The HepG2 cell line purchased from the KCLB (Korea Cells Line Bank, Republic of Korea) was cultured in complete DMEM. The cells were maintained in a 5% CO_2_ humidified incubator at 37 °C. The well-grown cells were harvested, trypsinized with Trypsin-EDTA, and 100 μL/well, were seeded into separate microplates at a density of 5 × 10^4^ cells per well. The viability of the HepG2 cells was counted as 95% as determined by the Trypan Blue exclusion. Cells were incubated in a humidified incubator at 37 °C containing 5% CO_2_ and 95% air for 24 h. After 24 h incubation, the media was removed, and the cell was exposed to different concentrations of the Fbf-AgNPs and Bbf-AgNPs and dispersed in complete DMEM for 24 h at 37 °C containing 5% CO_2_. The cell cytotoxicity of Fbf-AgNPs and Bbf-AgNPs exposed cells was determined by the EZ-cytox kit (DoGenBio Co., Ltd., Seoul, South Korea) following the company’s instructions. After 24 h of exposure, the supernatant was replaced with fresh complete media containing 10 μL of EZ-cytox solution (110 μL) and incubated for around 20 min till visualization of color changes to yellowish orange. After incubation, the samples (100 μL) were aliquoted in a new microplate without any disturbance, and absorbance was recorded at 450 nm using a spectrophotometer. The cell morphology and viability study of Fbf-AgNPs and Bbf-AgNPs exposed cells was determined by the trypan blue exclusion assay. Similarly, after 24 h of exposure, the supernatant was removed and the cells were washed quickly with DPBS (100 μL), followed by the addition of 20 μL of fresh complete DMEM and trypan blue mixture (1:1) to each well. The cell morphology and viability were observed under the inverted microscope (DMI6000B, Leica).

### 3.8. Statistical Analysis

The statistical analysis was done through ANOVA (one way) by following Duncan’s multiple tests (using SPSS; Software version 27.0 and IBM Crop, Armonk, NY, USA) at a 5% of significance level *p* < 0.05. Correlation and regression analysis was also carried out using the SPSS software.

## 4. Conclusions

The fern-mediated biosynthesis of silver nanoparticles is natural and economical, and is synthesized using both fresh and boiled fern extracts. In the synthesis process, the bioactive compounds existing in the green extracts play a substantial part in the capping and reduction of the nanoparticle. In the biological assays, individually both the obtained AgNPs demonstrated considerable α-glucosidase inhibition, antioxidant, and cytotoxicity potentials. In the α-glucosidase inhibition and cytotoxicity assays, Bbf-AgNPs displayed slightly higher effectiveness than the Fbf-AgNPs. In antioxidant assays both Fbf-AgNPs and Bbf-AgNPs displayed moderate effects. From the overall biological results, it can be said that, for this particular fern species, the boiled fern is slightly better and more effective than the fresh one. Both the fresh and boiled fern-mediated AgNPs with potential multitherapeutic effects could serve as a potential candidate for the cosmetics and pharmaceutical industries after intense safety investigations.

## Figures and Tables

**Table 1:** Preliminary phytochemical screening of Fbf and Bbf aqueous extracts.

Name of Phytochemicals	Fbf Aqueous Extract	Bbf Aqueous Extract
Tannin	+	-
Flavonoids	+	+
Terpenoids	-	+
Saponins	+	+
Steroids	+	-
Carbohydrates	+	+
Cardiac steroidal glycoside	-	+

+ = detected; and - = not detected.

**Table 2:** IC_50_ values of, Fbf-AgNPs and Bbf-AgNPs in the antioxidant, α-glucosidase inhibition, and cytotoxicity studies.

Parameters	IC_50_ Value (µg/mL) Fbf-AgNPs	IC_50_ Value (µg/mL) Bbf-AgNPs
α-glucosidase inhibition	1.56	1.44
Cytotoxicity	26.96	17.35
DPPH scavenging	72.59	82.56
ABTS scavenging	332.40	206.68
Reducing power (* IC_0.5_ value)	* 231.07	* 292.66

* is IC_0.5_.

## Data Availability

All data are presented as tables and figures in the manuscript.
